# Validity of Self-Reported Height, Weight, and Body Mass Index: Findings from the National Health and Nutrition Examination Survey, 2001-2006

**Published:** 2009-09-15

**Authors:** Ray M Merrill, John S Richardson

**Affiliations:** Brigham Young University, Department of Health Science; Brigham Young University, Provo, Utah

## Abstract

**Introduction:**

Our study extends previous studies that have evaluated the level of bias in self-reported height and weight and corresponding body mass index (BMI). Results are evaluated by age, sex, income, race/ethnicity, and BMI classifications.

**Methods:**

Analyses are based on the National Health and Nutrition Examination Survey (NHANES) from 2001-2006. The sample was 8,208 men and 8,606 women aged 16 years or older.

**Results:**

On average, men overreport their height by 1.22 cm (0.48 in) and their weight by 0.30 kg (0.66 lbs), and women overreport their height by 0.68 cm (0.27 in) and underreport their weight by −1.39 kg (−3.06 lbs). Overreporting of height significantly increases with age after age 50 for men and after age 60 for women. Overreporting of weight in men is significant in the age groups 16 to 49 years and 70 years or older. Women significantly underreport their weight in each age group but more so in the age group 16 to 49 years, followed by 50 to 69 years, and then 70 years or older. Men are more likely than women to think their weight is about right if they are at a normal weight or are overweight or obese, but women are more likely to think their weight is about right if they are underweight.

**Conclusion:**

Men and women significantly overreport their height, increasingly so at older ages. Men tend to overestimate their weight, but women underreport their weight, more so in younger ages. Corresponding BMI is underestimated, more so for women than for men at each age and increasingly so with older age for both sexes.

## Introduction

It is well established that excessive body weight is a risk factor for illness and death due to diabetes, stroke, coronary artery disease, hypertension, high cholesterol, and kidney and gallbladder disorders (1,2). Excessive body weight may also increase the risk of some types of cancer and the development of osteoarthritis and sleep apnea (3,4). Consequently, many public health interventions have been designed to assist people to maintain or attain a proper weight. Monitoring the efficacy of such programs often relies on self-reported weight and body mass index (BMI), which is a function of height and weight. It is assumed, therefore, that interventions tailored to a person's weight or BMI and evaluation of trends in weight or BMI are based on accurate measures of weight or BMI.

Previous studies have assessed the accuracy of self-reported height, weight, and corresponding BMI in adults (5-14). One study compared self-reported and measured height and weight of a large population in Canada (5). Self-reported height was found to be an average of 0.88 cm greater than measured height, and self-reported weight was 2.33 kg less than measured weight. Consequently, BMI derived from self-reported height and weight was 1.16 lower than BMI derived from measurements. In the same study, self-reported overweight was 31.9% compared with 33.7% measured overweight, and self-reported obesity was 15.3% compared with 22.9% measured obesity.

In the United States, Rowland used NHANES II to show that self-reported height was greater than measured, increasingly so among participants aged 45 years or older (12). He also studied the effects of age on weight and BMI status showing, for example, that severely overweight men and women aged 20 to 34 years underreported their weight significantly more than did those aged 55 to 74 years. Kuczmarski et al used NHANES III to assess the effect of age on the validity of self-reported height, weight, and BMI (13). They found that increasing age is generally associated with a greater overreporting bias for height and underreporting bias for weight and that the bias associated with self-reported weight in women decreases with older age.

Studies have investigated the correlation of bias associated with self-reported height, weight, and corresponding BMI with variables other than age. A study of NHANES III data showed that bias in self-reported weight was affected by race/ethnicity and education in addition to age and BMI (14). Furthermore, consumption of more than 100 cigarettes in a lifetime and a desire to change weight were predictors of bias for men, while marital status, income, activity level, and the number of months since the last doctor's visit were predictors for women (14). NHANES III data have also demonstrated that bias in self-reported weight was greater in Hispanics than in non-Hispanic whites, for both men and women (15). McAdams et al found differences between measured and self-reported height, weight, and BMI according to sex and race/ethnicity by analyzing NHANES III data; however, their major findings were significant correlations between self-reported measures and certain biomarkers such as fasting blood glucose, high-density lipoprotein cholesterol, and systolic blood pressure (16). Kuchler and Variyam used NHANES III to find that adults in the United States often misperceive their weight status (17). Finally, 2 other studies found that bias in self-reported weight and height were greater when using data from the telephone-based Behavioral Risk Factor Surveillance System than when using data from personal interviews conducted in NHANES (18,19).

Although these studies involving NHANES data identified the role of age, sex, weight, and race/ethnicity on bias in self-reported weight, all used data that were gathered before 1995. Bias in self-reported height and weight for people who are underweight or of normal weight according to age and sex deserves further attention. The level of reporting bias according to self-perceived weight has received little attention.

The purpose of this study is to confirm and extend previous studies that have evaluated the level of bias in self-reported height and weight by using cross-sectional NHANES data for 2001-2006. We assess the level of bias in self-reported height and weight and corresponding bias in BMI estimates. Because previous studies have shown the level of bias to be related to age, sex, race/ethnicity, and perception of one's weight (5,12-14,17), we evaluated bias in self-reported height and weight according to these variables.

## Methods

### Study population

We used data from participants aged 16 years or older from 3 cross-sectional NHANES surveys, covering 2001-2002, 2003-2004, and 2005-2006 (20-22).

### National Health and Nutrition Examination Survey

NHANES is a survey of the National Center for Health Statistics that assesses the health and nutritional status of children and adults in the United States. The survey combines interviews and physical examinations from a sample of the US noninstitutionalized civilian population. The interview includes questions about demographics, socioeconomic status, diet, and health. The examination component of the survey includes medical, dental, and physiological measures (23-25).

Health interviews are conducted in participants' homes using computer-assisted personal interviewing systems, and health measurements are performed in specifically designed mobile centers that are conveniently located at survey locations throughout the country. One physician and several medical and health technicians make up the study team. Body measurements are taken on all participants, and laboratory tests are administered. Staff members are generally bilingual, speaking both English and Spanish. The information collected in the survey is kept strictly confidential. NHANES received approval through the National Center for Health Statistics Ethics Review Board (26).

NHANES is a complex sample survey. The sampling frame for this design includes all counties in the United States. Clusters of households are selected, and each person in a household is screened for demographic characteristics. Then, 1 or more members of each household are selected for the sample. NHANES is designed to assist and encourage participation. For example, transportation is provided to the mobile centers, compensation is given, and a report of medical findings is provided to each participant. For the 3 NHANES data sets used in our study (2001-2002, 2003-2004, and 2005-2006), the interview response rates were 84%, 79%, and 80%, and the examination response rates were 80%, 76%, and 77%, respectively (27).

### Characteristic variables

Several body measurements were taken during the physical examination, including standing height (cm) and weight (kg), which were considered in this study. From these measurements BMI was determined. Demographic information considered was age, sex, annual household income, and race/ethnicity. Self-reported height and weight, and whether participants considered their weight to be about right, overweight, or underweight was obtained from the weight history module of the questionnaire.

Age in years was categorized as 16 to 29, 30 to 39, 40 to 49, 50 to 59, 60 to 69, 70 to 79, and 80 years or older; race/ethnicity was categorized as Hispanic, white non-Hispanic, black non-Hispanic, and other; and annual household income was categorized as less than $20,000; $20,000 to $34,999; $35,000 to $54,999; $55,000 to $74,999; and $75,000 or more. BMI (kg/m^2^) was categorized to determine weight status: underweight (<18.5), normal (18.5-24.9), overweight (25.0-29.9), and obese (≥30.0).

### Statistics

Sample weights, stratification, and clustering of the design were incorporated into the analyses to obtain unbiased national estimates and standard errors of estimates. Multiple regression analyses were used to simultaneously assess the effects of age, race/ethnicity, annual household income, and weight classification on reporting bias. All analyses were performed using the SAS/Stat 9.1 (SAS Institute Inc, Cary, North Carolina).

## Results

Self-reported and measured height, weight, and BMI for 16,814 participants (8,8,208 men and 8,606 women) were obtained according to calendar years. After adjusting for age and sex, the difference between self-reported and measured information did not significantly vary across calendar years for height (*P* = .16), weight (*P* = .31), or BMI (*P* = .35). The percentage of participants who perceived their weight as about right remained constant (40% in 2001-2002, 39% in 2003-2004, and 39% in 2005-2006; *P* = .92), despite an increase in measured BMI during these same time periods (27.2 kg/m^2^, 27.9 kg/m^2^, and 28.3 kg/m^2^, respectively). Therefore, analyses are based on the combined years 2001 through 2006.

On average, men overreported their height by 1.22 cm (0.48 in) and their weight by 0.30 kg (0.66 lbs), and women overreported their height by 0.68 cm (0.27 in) and underreported their weight by −1.39 kg (−3.06 lbs) (Table 1). BMI was underestimated for both men and women.

On average, both men and women significantly overreported their actual height, men more so than women (Table 2). Significant overreporting of height is greater among older men than younger men, among white and black non-Hispanic men than Hispanic men and men of other races/ethnicities, and among overweight and obese men than underweight or normal-weight men. Significant overreporting of height is greater among older women, Hispanic women, women in lower income categories, and overweight and obese women.

Self-reporting bias in weight significantly differs between men and women. Although men significantly overreport their weight in the age groups 16 to 39 years and 70 years or older, women significantly underreport their weight in the age range 16 to 79 years. Only white and black non-Hispanic men significantly overreport their weight, whereas all racial/ethnic groups for women significantly underreport their weight. Men in the income bracket $20,000 to $34,999 significantly overreport their weight, but women across all income categories significantly underreport their weight. Men in each BMI category significantly overreport their weight, with the exception of obese men, who significantly underreport their weight. Conversely, women who are normal weight, overweight, or obese significantly underreport their weight, increasingly so as BMI increases.

Self-reported height bias was significantly associated with age, sex, income, and BMI weight classification, but not with race/ethnicity. No significant interactions were found among these variables. Self-reported weight bias was also significantly associated with age, sex, income, and BMI weight classification, but not with race/ethnicity. Significant interactions were observed between age and sex (*P* = .01), age and income (*P* = .04), and age and BMI weight classification (*P* < .001). Significant overreporting of weight in men was found in the age groups 16 to 49 years and 70 years or older (Table 3). Conversely, women significantly underreported their weight in each age group. Underreporting of height is more pronounced in participants of higher income groups for people aged 50 to 69 years, but income has less of an influence on reporting bias in the younger or older age groups (data not shown). Underreporting of weight among people who are obese is significantly greater among people aged 16 to 49 years, followed by people aged 50 to 69 years, and then people aged 70 years or older.

Approximately 39% (44% men and 34% women) thought their weight was about right, approximately 5% (7% men and 3% women) thought they were underweight, and 56% (48% men and 63% women) thought they were overweight. The difference in responses between men and women was significant (*P* < .001). The percentage who thought their weight was about right according to their actual BMI weight classification is presented in the Figure. Men were more likely than women to think their weight was about right if they were normal weight, overweight, or obese. Women were more likely than men to think their weight was about right if they were underweight.

**Figure 1:**
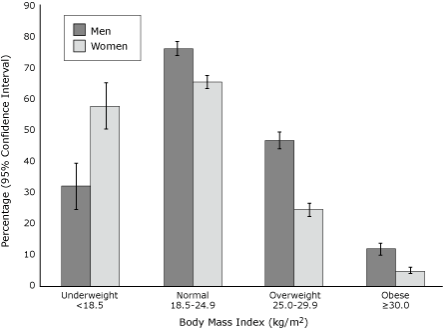
Participants Who Perceived Their Weight as About Right, By Sex and BMI Classification, National Health and Nutrition Examination Survey, 2001-2006.

**Table:** 

**Body Mass Index (kg/m^2^)**	**Sex**	
	**Male, % (95% CI)**	**Female, % (95% CI)**
Underweight (<18.5)	32.0 (24.7-39.4)	57.7 (50.3-65.1)
Normal weight (18.5-24.9)	76.2 (74.0-78.4)	65.5 (63.2-67.7)
Overweight (25.0-29.9)	46.8 (44.1-49.5)	24.4 (22.3-26.5)
Obese (≥30.0)	12.0 (10.1-13.8)	4.9 (3.9-5.9)

## Discussion

This article has described the level of bias in self-reported height, weight, and corresponding BMI. Men tended to overreport their height and weight, whereas women tended to overreport their height but underreport their weight. Consequently, BMI of both men and women is significantly underestimated. Had self-reported height and weight been obtained over the telephone rather than face-to-face, the bias would likely be more pronounced (18,19).

Lower self-reported height than measured height increased with age for both men and women, which has been observed previously (5,12,13). Loss of height during the aging process is normal for both men and women. This loss in height occurs because the discs that cushion and separate the vertebrae of the spine compress and shrink over time. Loss in height also occurs because of compression and deterioration of the vertebrae as a result of bone loss (ie, osteoporosis). People typically lose about 1 cm (0.4 in) every decade after age 40 (28). Greater overreporting of height with older age is consistent with people not being aware of the extent of their height loss as they age.

Elgar and Stewart found that both men and women underreported their weight, women more so across the age span. In contrast, our data show that men aged 16 to 39 years and aged 70 years or older significantly overreported their weight, but women significantly underreported their weight in each age group. Men in our study only significantly underreported their weight if they were obese. The underreporting of weight among women, but generally not among men, is consistent with women being less satisfied with their weight. McAdams et al found that self-reported weight for white non-Hispanic men was not significantly biased although self-reported weight of black and Hispanic men was (16). Our study found that white and black non-Hispanic men significantly overreported their weight, whereas Hispanic men did not. Furthermore, no difference was observed in corresponding bias in BMI among racial/ethnic groups for either men or women. In contrast, Gillium and Sempos, based on data from NHANES III, showed that self-reporting bias of BMI was greater in Hispanic men and women compared with white non-Hispanic men and women (15).

In this study, women underreported their weight similarly across all income groups. In contrast, men overreported weight in the lower income groups but not in the higher. Although it has not been previously considered, overreporting of height was associated with income in this study, with the level of overreporting of height decreasing as income increases. Using data from NHANES III, Villanueva similarly showed that men were more likely to overestimate their weight in lower income categories (14). However, this same study showed that underreporting of weight was significantly greater among women with annual incomes greater than $30,000 (14).

No previous study has focused on trends in overreporting height according to BMI classification. This study shows that overreporting bias in height increases significantly with weight classification. Conversely, studies have observed that a person's actual weight influences their self-reported weight; underreporting bias in self-reported weight increases with higher weight (12,29), as also demonstrated by this study.

Kuchler and Variyam analyzed NHANES III data to describe the percentage of men and women who perceived their weight as about right, according to weight status; however, they did not distinguish the amount of those who were underweight (17). Accordingly, they found that 43% of overweight men and 18% of overweight women thought their weight was about right. In our study, 47% of overweight men and 24% of overweight women thought their weight was about right. Therefore, a greater percentage of overweight men and women today may consider their weight to be about right, which may be a result of the growing acceptance of heavier weights.

Men are more likely than women to overreport their weight if they are actually underweight, whereas women are more likely than men to underreport their weight if they are overweight or obese. This finding is consistent with previous research (17). Men in general tend to feel better about their bodies, but among people who are underweight, women feel better about their weight than do men (30).

NHANES is a comprehensive national survey with high response rates and minimal missing data. Data are representative of a sample of the United States population, so results are generalizable. Oversampling of ethnic minorities also ensures that adequate numbers of minorities are available for analyses. Nevertheless, NHANES data are limited because the survey design is cross-sectional, so causal inferences cannot be made and confounding may be an issue. In addition, NHANES does not include institutionalized people, such as those in long-term care facilities who are not as healthy as or who are more likely to have functional limitations than the general population.

BMI is underestimated for both men and women for different reasons. On average, men significantly overreport their height and weight, and women significantly overreport their height and underreport their weight. Men are more likely than women to think their weight is about right if they are normal weight, overweight, or obese, but women are more likely to think their weight is about right if they are underweight. Assessment of reporting bias of height, weight, and corresponding BMI according to age, sex, income, and BMI classification indicates those categories where bias is high, low, or not present. Furthermore, BMI based on self-reported height and weight is underestimated for both men and women, increasingly so with older age and weight. Such information may be particularly useful to researchers as they evaluate the effects of BMI based on self-reported height and weight on disease outcomes according to selected subgroups.

## Figures and Tables

**Table 1:** Mean Difference Between Self-Reported and Measured Height, Weight, and Body Mass Index, by Sex, National Health and Nutrition Examination Survey, 2001-2006^a^

Characteristic	Self-Reported	Measured	Mean Difference (95% CI)
**Men**			
Standing height, cm	177.67	176.45	1.22 (1.15, 1.28)
Weight, kg	87.55	87.25	0.30 (0.20, 0.40)
Body mass index (kg/m^2^)	27.62	27.96	−0.34 (−0.38, −0.30)
**Women**			
Standing height, cm	162.99	162.31	0.68 (0.62, 0.74)
Weight, kg	72.46	73.85	−1.39 (−1.48, −1.30)
Body mass index (kg/m^2^)	27.20	28.02	−0.82 (−0.85, −0.77)

Abbreviation: CI, confidence interval.

a Data source: NHANES, 2001-2006. Estimates were weighted to produce unbiased national estimates.

**Table 2:** Mean Difference Between Self-Reported and Measured Height, Weight, and BMI in Men and Women, by Selected Characteristics^a^

Characteristic	No. of Participants	Height, cm, Mean 95% CI	Weight, kg, Mean (95% CI)	BMI, kg/m^2^, Mean (95% CI)

**Men**				

**Age, y**				
16-29	2800	**0.97** (0.77, 1.16)	**0.37** (0.07, 0.68)	**−0.21** (−0.33, −0.08)
30-39	1076	**0.91** (0.70, 1.11)	**0.34** (0.07, 0.60)	**−0.23** (−0.35, −0.12)
40-49	1193	**0.75** (0.57, 0.93)	0.21 (−0.12, 0.53)	**−0.24** (−0.35, −0.13)
50-59	912	**1.20** (1.02, 1.38)	0.07 (−0.19, 0.33)	**−0.42 **(−0.53, −0.32)
60-69	964	**1.94** (1.71, 2.17)	0.24 (−0.03, 0.51)	**−0.63 **(−0.74, −0.51)
70-79	788	**2.54** (2.28, 2.80)	**0.57** (0.34, 0.81)	**−0.66** (−0.75, −0.57)
≥80	475	**3.88** (3.55, 4.21)	**1.10** (0.80, 1.40)	**−0.85** (−1.01, −0.69)
**Race/ethnicity**				
Hispanic	2011	**0.70** (0.47, 0.93)	0.10 (−0.19, 0.38)	**−0.22** (−0.33, −0.11)
White, non-Hispanic	3942	**1.34** (1.21, 1.47)	**0.33** (0.15, 0.50)	**−0.37** (−0.43, −0.31)
Black, non-Hispanic	1953	**1.18** (1.02, 1.35)	**0.55 **(0.16, 0.95)	**−0.24** (−0.38, −0.10)
Other	302	**0.66** (0.25, 1.08)	−0.16 (−0.75, 0.43)	**−0.30** (−0.57, −0.03)
**Annual household income, $**				
<20,000	1650	**1.21 **(0.91, 1.51)	0.75 (0.34, 1.15)	**−0.16** (−0.34, 0.02)
20,000-34,999	1761	**1.55 **(1.34, 1.75)	**0.69** (0.42, 0.95)	**−0.31** (−0.40, −0.22)
35,000-54,999	1635	**1.35** (1.14, 1.55)	0.26 (−0.01, 0.53)	**−0.40** (−0.50, −0.30)
55,000-74,999	946	**1.19** (0.95, 1.43)	0.08 (−0.26, 0.43)	**−0.42** (−0.53, −0.32)
≥75,000	1737	**0.95** (0.82, 1.08)	0.05 (−0.21, 0.31)	**−0.34** (−0.44, −0.24)
**BMI, kg/m^2^**				
Underweight (<18.5)	273	**0.87** (0.47, 1.26)	**3.71 **(3.03, 4.39)	**0.97** (0.73, 1.21)
Normal (18.5-24.9)	2695	**0.85** (0.68, 1.02)	**1.69** (1.50, 1.87)	**0.29** (0.20, 0.38)
Overweight (25.0-29.9)	3055	**1.29** (1.17, 1.42)	**0.29** (0.11, 0.47)	**−0.35 **(−0.41, −0.29)
Obese (≥30.0)	2185	**1.50** (1.35, 1.64)	**−1.32** (−1.65, −1.00)	**−1.05** (−1.16, −0.94)

**Women**				

**Age, y**				
16-29	3097	**0.51** (0.38, 0.64)	**−1.99** (−2.23, −1.74)	**−0.94** (−1.04, −0.85)
30-39	1260	**0.50** (0.32, 0.68)	**−1.45** (−1.76, −1.15)	**−0.77** (−0.90, −0.64)
40-49	1163	0.12 (−0.03, 0.30)	**−1.36** (−1.70, −1.01)	**−0.62** (−0.77, −0.47)
50-59	885	**0.47** (0.31, 0.63)	**−1.41** (−1.74, −1.08)	**−0.78** (−0.92, −0.64)
60-69	994	**1.13** (0.91, 1.34)	**−1.00** (−1.25, −0.76)	**−0.85** (−0.97, −0.72)
70-79	653	**1.69** (1.37, 2.01)	**−0.55** (−0.76, −0.34)	**−0.85 **(−0.97, −0.73)
≥80	554	**3.09** (2.73, 3.46)	−0.13 (−0.44, 0.17)	**−1.08** (−1.24, −0.93)
**Race/ethnicity**				
Hispanic	2099	**1.18** (0.88, 1.47)	**−1.16** (−1.43, −0.89)	**−0.91** (−1.08, −0.73)
White, non-Hispanic	4154	**0.56** (0.46, 0.67)	**−1.38** (−1.53, −1.23)	**−0.76** (−0.83, −0.70)
Black, non-Hispanic	1991	**0.79** (0.65, 0.92)	**−1.69** (−2.03, −1.35)	**−0.99** (−1.12, −0.85)
Other	362	**1.03** (0.67, 1.38)	**−1.29** (−1.67, −0.92)	**−0.87** (−1.06, −0.68)
**Annual household income, $**				
<20,000	2080	**1.23** (1.06, 1.41)	**−1.48** (−1.73, −1.24)	**−1.05** (−1.15, −0.95)
20,000-34,999	1765	**0.9** (0.71, 1.08)	**−1.11** (−1.35, −0.87)	**−0.77** (−0.88, −0.66)
35,000-54,999	1539	**0.58** (0.38, 0.79)	**−1.55** (−1.89, −1.22)	**−0.83** (−0.98, −0.69)
55,000-74,999	969	**0.49** (0.31, 0.67)	**−1.46** (−1.82, −1.09)	**−0.77** (−0.93, −0.61)
≥75,000	1715	**0.28 **(0.13, 0.43)	**−1.41** (−1.64, −1.18)	**−0.67 **(−0.78, −0.57)
**BMI, kg/m^2^**				
Underweight (<18.5)	371	**0.36** (0.08, 0.64)	**1.16 **(0.88, 1.44)	**0.32** (0.22, 0.44)
Normal (18.5-24.9)	2925	**0.35** (0.23, 0.47)	**−0.2** (−0.30, −0.09)	**−0.2** (−0.26, −0.15)
Overweight (25.0-29.9)	2459	**0.89** (0.73, 1.06)	**−1.46** (−1.68, −1.23)	**−0.88** (−0.97, −0.79)
Obese (≥30.0)	2851	**0.92** (0.77, 1.07)	**−2.99 **(−3.30, −2.68)	**−1.58 **(−1.70, −1.46)

Abbreviations: BMI, body mass index; CI, confidence interval.

a Data source: NHANES, 2001-2006. Estimates were weighted to produce unbiased national estimates. Bolded items are significant (*P* < .05) across the levels of the respective variables.

**Table 3:** Mean Difference Between Self-Reported and Measured Weight (kg), by Selected Variables^a^

Characteristic	Age 16-49 y, Mean (95% CI)	Age 50-69 y, Mean (95% CI)	Age ≥70 y, Mean (95% CI)
**Sex**			
Male	**0.31 **(0.10, 0.52)	0.13 (−0.06, 0.33)	**0.73 **(0.52, 0.93)
Female	**−1.63** (−1.80, −1.46)	**−1.25** (−1.47, −1.03)	**−0.39** (−0.56, −0.23)
**Annual household income, $**			
<20,000	**−0.85** (−1.20, −0.50)	−0.30 (−0.65, 0.04)	0 (−0.30, 0.30)
20,000-34,999	**−0.46** (−0.70, −0.23)	−0.21 (−0.61, 0.18)	**0.35** (0.02, 0.69)
35,000-54,999	**−0.83** (−1.10, −0.55)	**−0.40** (−0.73, −0.08)	0.05 (−0.27, 0.36)
55,000-74,999	**−0.59** (−0.89, −0.29)	**−0.98** (−1.40, −0.56)	−0.38 (−0.78, 0.02)
≥75,000	**−0.60** (−0.80, −0.41)	**−0.84** (−1.07, −0.60)	−0.22 (−0.64, 0.20)
Not reported	**−0.81** (−1.27, −0.36)	**−0.63** (−1.15, −0.11)	0.29 (−0.18, 0.76)
**Body mass index (kg/m^2^)**			
Underweight (<18.5)	**2.25** (1.90, 2.60)	**1.21** (0.08, 2.34)	**1.77** (1.26, 2.27)
Normal (18.5-24.9)	**0.58** (0.42, 0.75)	**0.59** (0.41, 0.78)	**0.90** (0.63, 1.16)
Overweight (25.0-29.9)	**−0.65** (−0.83, −0.48)	**−0.20** (−0.40, −0.01)	−0.04 (−0.21, 0.14)
Obese (≥30.0)	**−2.61** (−2.94, −2.29)	**−1.81** (−2.14, −1.48)	**−0.92** (−1.22, −0.63)

Abbreviation: CI, confidence interval.

a Data source: NHANES, 2001-2006. Estimates were weighted to produce unbiased national estimates. Bolded items are significant (*P* < .05) across the levels of the respective variables.
